# Active Components with Inhibitory Activities on IFN-γ/STAT1 and IL-6/STAT3 Signaling Pathways from Caulis Trachelospermi

**DOI:** 10.3390/molecules190811560

**Published:** 2014-08-05

**Authors:** Xiao-Ting Liu, Zhe-Xing Wang, Yu Yang, Lin Wang, Ruo-Feng Sun, Yi-Min Zhao, Neng-Jiang Yu

**Affiliations:** 1 School of Traditional Chinese Materia Medica, Shenyang Pharmaceutical University, Shenyang 110016, China; 2 Beijing Institute of Pharmacology and Toxicology, Beijing 100850, China

**Keywords:** Caulis Trachelospermi, 4-demethyltraxillaside, nortrachelogenin 4-*O*-*β*-d-glucopyranoside, JAK/STAT signaling pathway

## Abstract

Initial investigation for new active herbal extract with inhibiting activity on JAK/STAT signaling pathway revealed that the extract of Caulis Trachelospermi, which was separated by 80% alcohol extraction and subsequent HP-20 macroporous resin column chromatography, was founded to strongly inhibit IFN-γ-induced STAT1-responsive luciferase activity (IFN-γ/STAT1) with IC_50_ value of 2.43 μg/mL as well as inhibiting IL-6-induced STAT3-responsive luciferase activity (IL-6/STAT3) with IC_50_ value of 1.38 μg/mL. Subsequent study on its active components led to the isolation and identification of two new dibenzylbutyrolactone lignans named 4-demethyltraxillaside (**1**) and nortrachelogenin 4-*O*-*β-*d-glucopyranoside (**2**), together with six known compounds. The lignan compounds **1**–**4** together with other lignan compounds isolated in previous study were tested the activities on IFN-γ/STAT1 and IL-6/STAT3 pathways. The following result showed that the main components trachelogenin and arctigenin had corresponding activities on IFN-γ/STAT1 pathway with IC_50_ values of 3.14 μM and 9.46 μM as well as trachelogenin, arctigenin and matairesinol strongly inhibiting IL-6/STAT3 pathway with IC_50_ values of 3.63 μM, 6.47 μM and 2.92 μM, respectively.

## 1. Introduction

**Figure 1:**
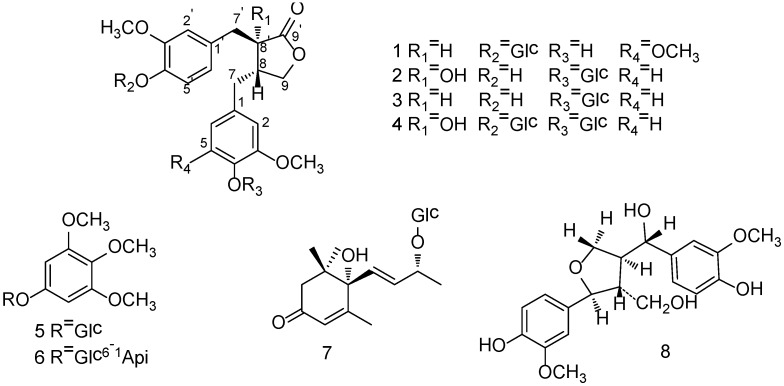
Chemical structures of compounds **1**–**8**.

Caulis Trachelospermi, the stems and leaves of *Trachelospermum jasminoides* (Lindl.) Lem, is mainly distributed in Henan, Anhui, Hubei, Shandong and Guangxi provinces in China. It has been used in Traditional Chinese Medicine for the treatment of rheumatic arthralgia, aching of the loins and knees, traumatic injuries [1] and its medicinal values such as anticancer, anti-inflammation had been reported [2,3]. Chemical investigations indicated that it mainly contained lignans, flavonoids and triterpenoids [4,5,6]. In our previous study, the extract of Caulis Trachelospermi, which was separated by 80% alcohol extraction and subsequent HP-20 macroporous resin column chromatography, exhibited marked anti-inflammatory activity in animal model [7] as well as moderate inhibiting activity on NF-*κ*B signaling pathway induced by TNF-α [8], its active chemical investigation led to the discovery of 30 compounds including 19 dibenzylbutyrolactone lignans [7,8,9,10,11] and its main components, trachelogenin, nortrachelogenin and matairesinol, showed corresponding moderate inhibiting activities on TNF-α/NF-*κ*B pathway [8]. The JAK (Janus kinase)/STAT (signal transducer and activator of transcription) pathway is another signaling pathway responsible for signal transduction of a large number of cytokines [12]. Constitutive activation of the JAK/STAT pathway is frequently associated with cancer and autoimmune diseases, and it has been considered as an important target for therapeutic intervention [13,14,15]. Our recent study revealed the extract of Caulis Trachelospermi and its main components trachelogenin, arctigenin and matairesinol having strong inhibiting activity on the pathway, which may be an imprtant mechanism for the anticancer and anti-inflammation function of Caulis Trachelospermi. To further investigate active components, a phytochemical study guided by HPLC finger print chromatography [16] was performed and two new dibenzylbutyrolactone lignans named 4-demethyltraxillaside (**1**) and nortrachelogenin 4-*O*-*β-*d-glucopyranoside (**2**), together with six known compounds (Figure 1) were isolated. Further screening for active compounds resulted that three main dibenzylbutyrolatone lignan components trachelogenin, actigenin and matairesinol had corresponding strong activities on the JAK/STAT pathway. In this paper, we report the isolation and structural elucidation of compounds **1** and **2** as well as the JAK/STAT pathway inhibiting activities of the extract and lignan components. Spectral data of the known compounds **3** and **4** whose detailed NMR data has not been reported to date is also described.

## 2. Results and Discussion

An 80% alcohol extract of Caulis Trachelospermi was applied on a HP-20 macroporous resin column and eluted with water and 70% alcohol successively. The 70% alcohol elution was subjected to multiple chromatographic techniques using Sephadex LH-20, ODS column chromatography (CC) and semi-preparative RP-HPLC to furnish two new lignans and six known compounds.

**Figure 2:**
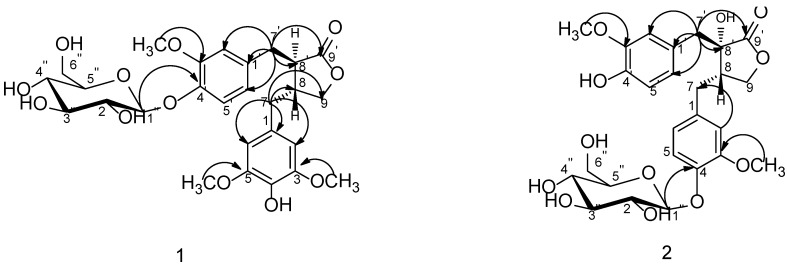
Key HMBC correlations of compounds **1** and **2**.

Compound **1** was obtained as white amorphous powder with the molecular formula C_2__7_H_3__4_O_1__2_ on the basis of HRESIMS (*m/z* 573.1945 [M+Na]^+^, calcd. 573.1942). ^1^H-NMR (Table 1) spectrum displayed signals for an ABX system assignable to 3,4-disubstituted phenyl group at *δ* 6.99 (1H, d, *J* = 8.3 Hz, H-5'), 6.68 (1H, dd, *J* = 8.3, 1.8 Hz, H-6') and 6.80 (1H, d, *J* = 1.8 Hz, H-2'), a singlet at *δ* 6.34 (2H, s, H-2 and H-6) to present a 3,4,5-trisubstituted phenyl group, an oxygenated methylene at *δ* 4.11 (1H, dd, *J* = 8.5, 7.2 Hz, Ha-9) and 3.89 (1H, t, *J* = 8.5 Hz, Hb-9), two methylenes at *δ* 2.45–2.52 (2H, m, H-7) and 2.80–2.83 (2H, m, H-7'), two methines at *δ* 2.40–2.45 (1H, m, H-8) and 2.75 (1H, dd, *J* = 8.2, 6.1 Hz, H-8'). ^13^C-NMR spectrum which was sorted by HMQC data showed the presence of a carbonyl carbon, twelve aromatic carbons including seven quaternary carbons and five tertiary carbons, an oxygenated methylene carbon, four high-field carbons including two methylene carbons and two methine carbons. The spectral data mentioned above is characteristic to dibenzylbutyrolactone lignan. HMBC correlations from C-9' (*δ* 178.6) to H-7' as well as that from H-7' to three aromatic carbons at *δ* 131.9 (C-1'), 113.9 (C-2'), 121.4 (C-6') indicated that the 3,4-disubstituted phenyl group was connected with C-7'. So, the 3,4,5-trisubstituted phenyl group was deduced the linkage with C-7 which was also proved by HMBC correlations as shown in Figure 2. Carbon signals at *δ* 131.9, 113.9, 148.7, 145.4, 115.1, 121.4 were assigned to C-1'–6' as well as signals at *δ* 128.8, 106.0, 148.0, 133.9, 148.0, 106.0 were assigned to C-1–6. The ^1^H-NMR spectrum also showed three methoxyl groups at *δ* 3.71 and 3.72 which exhibited respective HMBC correlations with carbons at *δ* 148.0 (C-3, C-5) and 148.7 (C-3'), a phenolic hydroxy at *δ* 8.17 (1H, s, 4-OH), together with the presence of a sugar anomeric proton at *δ* 4.84 (1H, d, *J* = 7.3 Hz). Accordingly, Compound **1** was considered to be the glycoside of 4-demethyltraxillagenin which had been isolated from Caulis Trachelospermi [9]. HMBC correlation between the sugar anomeric hydrogen signal (*δ* 4.84) and C-4' (*δ* 145.4) implied that the sugar moiety was connected to C-4'. The diagnostic ^13^C-NMR spectral data of the sugar moiety and coupling constant of the anomeric proton (*J* = 7.3 Hz) indicated the sugar moiety was *β*-d-glucopyranosyl [4,17]. Its identical carbon chemical shifts with those of 4-demethyltraxillagenin indicated its absolute configuration was 8*R*, 8'*R* or 8*S*, 8'*S*. Compound **1** had negative cotton effects at 233 (Δε −2.72) and 276 nm (Δε −0.58) in CD spectrum, which determined its 8*R*, 8'*R* configuration according to the report that 8*R*,8'*R*-isomer has negative cotton effects at 233 and 276 nm in contrast to the positive cotton effects of 8*S*,8'*S*-isomer [18]. Consequently, the structure of **1** was identified as (8*R*,8'*R*)-4-hydroxy-3,3',4-trimethoxylignan-9,9'-olide-4'-*O*-*β*-d-glucopyranoside, namely 4-demethyltraxillaside.

**Table 1:** ^1^H-NMR (400 MHz) and ^13^C-NMR (100 MHz) spectral data of compounds **1** and **2** (in DMSO-*d*_6_, *δ* in ppm,*J* in Hz).

Position	1		2	
	*δ*_C_	*δ*_H_	*δ*_C_	*δ*_H_
1	128.8		133.0	
2	106.0	6.34 (1H, s)	113.0	6.70 (1H, d, 1.6)
3	148.0		148.8	
4	133.9		145.1	
5	148.0		115.3	6.97 (1H, d, 8.3)
6	106.0	6.34 (1H, s)	120.5	6.61 (1H, br d, 8.3)
7	37.3	2.45–2.52 (2H, m)	30.9	2.61 (1H, dd, 12.3, 2.4)
				2.44 (1H, br d, 12.3)
8	40.9	2.40–2.45 (1H, m)	42.8	2.39 (1H, m)
9	70.8	4.11 (1H, dd, 8.5, 7.2)	70.0	3.95 (2H, d, 7.7)
		3.89 (1H, t, 8.5)		
1'	131.9		126.4	
2'	113.9	6.80 (1H, d, 1.8)	114.5	6.77 (1H, br s)
3'	148.7		147.2	
4'	145.4		145.4	
5'	115.1	6.99 (1H, d, 8.3)	115.3	6.68 (1H, d, 8.0)
6'	121.4	6.68 (1H, dd, 8.3, 1.8)	122.7	6.61 (1H, br d, 8.0)
7'	33.6	2.80–2.83 (2H, m)	40.0 (overlapped)	2.98 (1H, d, 13.8)
				2.83 (1H, d, 13.8)
8'	45.6	2.75 (1H, dd, 8.2, 6.1)	75.4	
9'	178.6		178.1	
1''	100.3	4.84 (1H, d, 7.3)	100.2	4.82 (1H, d, 7.2)
2''	73.3		73.2	
3''	77.0		77.0	
4''	69.7		69.7	
5''	76.9		76.9	
6''	60.7		60.7	
4-OH		8.17 (1H, s)		
4'-OH				8.85 (1H, s)
8'-OH				6.21 (1H, s)
3-OCH_3_	56.0	3.71 (3H, s)	55.6	3.70 (3H, s)
3'-OCH_3_	55.7	3.72 (3H, s)	55.6	3.73 (3H, s)
5-OCH_3_	56.0	3.71 (3H, s)		

Compound **2** was isolated as white amorphous powder and its molecular formula was deduced as C_26_H_32_O_12_from HRESIMS data which showed a [M+Na]^+^ ion at *m/z* 559.1788 (calcd. 559.1786). ^1^H-NMR and ^13^C-NMR spectral data (Table 1) suggested it was also a dibenzylbutyrolactone lignan compound. The presence of a diagnostic hydroxyl signal at *δ*_H_ 6.21 and an AB-type coupled protons at *δ* 2.98, 2.83 (each 1H, d, *J* = 13.8 Hz, H-7') in ^1^H-NMR spectrum suggested compound **2** was a 8'-hydroxydibenzylbutyrolactone lignan. ^1^H-NMR spectrum also displayed signals of two sets of ABX systems of the phenyl protons to present two 3,4-disubstituted phenyl groups. The presence of two methoxy groups at *δ* 3.70 and 3.73 which had respective HMBC correlation with the carbon at *δ* 148.8 (C-3) and carbon at *δ* 147.2 (C-3') as well as a phenolic hydroxy at *δ* 8.85 (1H, s, 4'-OH) suggested the aglycone of **2** was nortrachelogenin which was proved by HMBC correlations as shown in Figure 2. A sugar anomeric proton at *δ* 4.82 (H-1'') with coupling constant of 7.2 Hz in ^1^H-NMR spectrum and its corresponding spectral data in ^1^^3^C-NMR spectrum indicated the presence of a *β*-d-glucopyranosyl group. HMBC correlation between the anomeric proton (H-1'') and carbon at *δ* 145.1 (C-4) or *δ* 145.4 (C-4') implied that the glucopyranosyl group was connected to C-4 or C-4'. According to compound **2** having evident different NMR spectral data from that of nortracheloside which had been isolated from Caulis Trachelospermi in our previous research, it was determined that the location of *β*-d-glucopyranoside was at C-4. Concerned the *β* effect of 8'-OH, optical isomers of 8'-hydroxydibenzylbutyrolactone lignan produced different chemical shifts at C-7' and C-8. *Trans* isomers were at about *δ* 42.0 and *δ* 43.7, but *cis* isomers were at about *δ* 38.3 and *δ* 48.1. Moreover, 8*S*-configuration produced negative optical rotation in contrast with positive optical rotation of its epimer [19]. Compound **2** produced similar C-7' and C-8 signals at *δ* 40.0, 42.7 and negative optical rotation 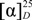 −48.5 (*c* 0.19, MeOH) revealed its absolute configuration was 8*S*, 8'*S*. Compound **2** was accordingly assigned as nortrachelogenin 4-*O*-*β*-d-glucopyranoside, structurally (8*S*,8'*S*)-4',8'-dihydroxy-3,3'-dimethoxylignan-9,9'-olide-4-*O*-*β*-d-glucopyranoside. A publication of patent application has concerned the same planar structure of **2** by covering with common structures [20], but no data of the production (isolation or synthesis) and structure elucidation was provided. Therefore, we report herein **2** as a new compound.

The known compounds **3**–**8** were identified as matairesinol 4-*O-β*-d-glucopyranoside (**3**) [21,22], nortrachelogenin 4,4'-di-*O-β*-d-glucopyranoside (**4**) [23,24], 3,4,5-trimethoxyphenol 1-*O-β*-d-glucopyranoside (**5**) [25,26], kelampayoside A (**6**) [27], roseoside (**7**) [28] and tanegool (**8**) [29], respectively, by comparing spectroscopic data with literature values.

Initial investigation for new active herbal extract with inhibiting activity on JAK/STAT signaling pathway revealed that the extract of Caulis Trachelospermi, which was separated by 80% alcohol extraction and subsequent HP-20 macroporous resin column chromatography, was founded to strongly inhibit IFN-γ-induced STAT1-responsive luciferase activity (IFN-γ/STAT1) with IC_50_ value of 2.43 μg/mL as well as inhibiting IL-6-induced STAT3-responsive luciferase activity (IL-6/STAT3) with IC_50_ value of 1.38 μg/mL *in vitro* (Table 2). In subsquent screening for high active components from the extract, lignan compounds **1**–**4** together with other lignan compounds isolated in previous study [7,8,9,10] were first tested in concentration of 5 μM which was close to the IC_50_ value of the extract. Following result showed aglycone compounds trachelogenin and arctigenin had high inhibition ratio more than 30% (77.5% and 32.6%) on IFN-γ/STAT1 pathway as well as aglycone compounds trachelogenin, arctigenin and matairesinol inhibiting IL-6/STAT3 pathway with inhibition ratio of 96.1%, 44.2% and 89.8%, respectively. Other aglycone compounds nortrachelogenin, 4-demethyltraxillagenin and 5-methoxytrachelogenin had low activity on IL-6/STAT3 pathway with inhibition ratio lower than 30% (Table 3). The activities of trachelogenin, arctigenin and matairesinol were further confirmed by IC_50_ evaluation. IC_50_ values of trachelogenin and arctigenin on IFN-γ/STAT1 pathway were 3.14 μM and 9.46 μM as well as IC_50_ values of trachelogenin, arctigenin and matairesinol on IL-6/STAT3 pathway were 3.63 μM, 6.47 μM and 2.92 μM (Table 2). Since signaling pathways are activated and deactivated within minutes after treatment. However, the luciferase assay in this study was performed after six hours of treatment. Therefore, the inhibition of the luciferase activity in this study may also be an indirect effect via the upregulation of the expression of other factors that then inhibit IFN-γ/STAT1 and IL-6/STAT3 signaling pathways.

**Table 2:** IC_50_ values of the extract and active compounds from Caulis Trachelospermi on IFN-γ/STAT1 and IL-6/STAT3 pathway.

Sample	IFN-γ/STAT1	IL-6/STAT3
extract	2.43 μg/mL	1.38 μg/mL
trachelogenin	3.14 μM	3.63 μM
arctigenin	9.46 μM	6.47 μM
matairesinol	-	2.92 μM
Pyridone 6 ^a^	0.0049 μM	-
AG490 ^a^	-	>100 μM

^a^ Positive control.

**Table 3:** Inhibition ratio (IR%) values of lignan components from Caulis Trachelospermi on IFN-γ/STAT1 and IL-6/STAT3 pathway.

Compound	Conc. (μM)	IFN-γ/STAT1	IL-6/STAT3
trachelogenin	5	77.5%	96.1%
tracheloside	5	9.4%	13.5%
trachelogenin 4'- *O*-*β*-gentiobioside	5	−8.0%	−17.5%
nortrachelogenin	5	−28.9%	27.3%
nortracheloside	5	−4.2%	2.3%
nortrachelogenin 8'- *O*-*β-*d-glucoside	5	−7.4%	1.6%
nortrachelogenin 5'- *C*-*β-*d-glucoside	5	−22.1%	−9.3%
nortrachelogenin 4- *O*-*β-*d-glucopyranoside (**2**)	5	−3.5%	10.9%
nortrachelogenin 4,4'-di- *O-β-*d-glucopyranoside (**4**)	5	−2.7%	−14.7%
arctigenin	5	32.6%	44.2%
arctiin	5	15.4%	−14.7%
matairesinol	5	7.2%	89.8%
matairesinoside	5	−9.4%	13.5%
matairesinol 4- *O-β*-d-glucopyranoside (**3**)	5	5.8%	7.0%
matairesinol 4'- *O*-*β*-gentiobioside	5	−12.0%	−12.9%
traxillagenin	5	−1.9%	−26.1%
traxillaside	5	−5.1%	−19.8%
4-demethyltraxillagenin	5	7.1%	22.4%
4-demethyltraxillaside (**1**)	5	6.3%	−10.5%
5-methoxytrachelogenin	5	−0.2%	14.3%
5-methoxytracheloside	5	−21.0%	4.8%

Otherwise, all glycosyl or disaccharosyl compounds had no visible activities on IFN-γ/STAT1 or IL-6/STAT3 pathway in concentration of 5 μM (Table 3). In our previous research, trachelogenin and its glycoside tracheloside could be detected in rat plasma after oral administration of the extract of Caulis Trachelospermi [30]. Moreover, Nose *et al.* reported that tracheloside and arctiin could be converted to their aglycone trachelogenin and actigenin in rat large intestinal flora [31]. Therefore, the lignan glycosides may have effects *in vivo* on the JAK/STAT pathway via conversion to their aglycone in the intestinal tract.

## 3. Experimental Section

### 3.1. General

Optical rotations were obtained on an Optical Activity Limited polAAr 3005 spectropolarimeter. CD spectra were recorded on a Biologic M450 spectropolarimeter. 1D- and 2D-NMR experiment were performed on a JEOL JNM-GX 400 NMR spectrometer with TMS as internal standard. ESIMS and HRESIMS were conducted on an Applied Biosystems API 3000 LC-MS spectrometer and an Agilent 6520 Q-TOF LC-MS spectrometer, respectively. Semi-preparative RP-HPLC was carried out with Shimadzu instrument equipped with LC-15C pump and SPD-15C detector using an YMC C_18_ column (5 μm, 10 × 250 mm; YMC Co. Ltd., Kyoto, Japan). HP-20 macroporous resin (Mitsubishi Chemical Co., Tokyo, Japan), ODS (50 μm, YMC Co. Ltd., Kyoto, Japan) and Sephadex™ LH-20 (GE Healthcare, Uppsala, Sweden) were employed for column chromatography (CC).

### 3.2. Plant and Compound Materials

Caulis Trachelospermi (the stems and leaves of *T. jasminoides* (Lindl.) Lem) were purchased from Beijing Qijing Chinese Herbs Factory, Beijing, in 2008 and authenticated by senior engineer Qiyun Ma, Beijing Institute of Pharmacology and Toxicology, Beijing, China. A voucher specimen (No. 20080130) has been deposited at the Department of Natural Products Chemistry, Beijing Institute of Pharmacology and Toxicology, Beijing, China. Compounds trachelogenin, tracheloside, trachelogenin 4'-*O*-*β*-gentiobioside, nortrachelogenin, nortracheloside, nortrachelogenin 8'-*O*-*β*-d-glucoside, nortrachelogenin 5'-*C*-*β*-d-glucoside, arctigenin, arctiin, matairesinol, matairesinoside, matairesinol 4'-O-*β*-gentiobioside, traxillagenin, traxillaside, 4-demethyltraxillagenin, 5-methoxytrachelogenin and 5-methoxytracheloside for the active tests were isolated and identified in our previous research [7,8,9,10].

### 3.3. Extraction and Isolation

Caulis Trachelospermi (160 kg) was extracted two times with 80% alcohol at boiling temperature. The extract was concentrated and diluted in 1600 L 5% alcohol. The solution was first centrifuged to remove the insoluble substance and then was passed through a HP-20 macroporous resin column (100 L) and eluted by 500 L water and 500 L 70% alcohol successively. The 70% alcohol elution was concentrated and dried to produce 5.3 kg product.

Guided by HPLC detection, the product (50 g) was applied on column chromatography over ODS eluted with MeOH–H_2_O (4:6) to afford seven fractions (A–G). **Fr-A** was separated over ODS CC eluted with a gradient 10% MeOH, 20% MeOH, 30% MeOH, 100% MeOH and eight fractions were collected. **Fr-A-3** was subjected to ODS CC eluted with MeOH–H_2_O (15:85) and then purified by semi-preparative RP-HPLC with mobile phase MeOH–H_2_O (16:84), to yield compounds **5** (11.4 mg) and **6** (24.0 mg). **Fr-A-4** was rechromatographed on an ODS column eluted with MeOH–H_2_O (2:8), followed by semi-preparative RP-HPLC [MeOH–H_2_O (17:83)] to give compound **8** (38.5 mg). **Fr-A-5**was fractionated by ODS CC eluted with MeOH–H_2_O (25:75) and then purified by preparative RP-HPLC [MeOH–H_2_O (17:83)] to yield compound **4** (45.0 mg). **Fr-A-6** was further isolated by ODS CC eluted with MeOH–H_2_O (3:7) then purified by Sephadex LH-20 (MeOH) and semi-preparative RP-HPLC [MeOH–H_2_O (2:8)] to afford compound 7 (1.9 mg). **Fr-A-8** was chromatographed with identical method of **Fr-A-6** to yield compound **2** (8.0 mg). **Fr-C** was fractionated by ODS CC eluted with MeOH–H_2_O (4:6) and seven fractions were obtained. **Fr-C-4** was chromatographed over ODS CC [MeOH–H_2_O (25:75)] and purified by semi-preparative RP-HPLC [MeOH–H_2_O (28:72)] to afford compounds **1** (15.7 mg) and **3** (7.8 mg).

### 3.4. Spectral Data

*4-Demethyltraxillaside* (**1**). White amorphous powder. 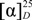 −44.1 (*c* 0.52, MeOH). CD (MeOH) λ_max_ nm (∆ε): 233 (−2.72), 276 (−0.58). ESIMS: *m/z* 573 [M+Na]^+^ (Pos.), 549 [M−H]^−^ (Neg.). HRESIMS: *m/z*: 573.1945 [M+Na]^+^ (Calcd. for C_27_H_34_O_12_Na, 573.1942). ^1^H-NMR and ^13^C-NMR data see Table 1.

*Nortrachelogenin 4-O-β-d-glucopyranoside* (**2**). White amorphous powder. 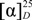 −48.5 (*c* 0.19, MeOH). CD (MeOH) λ_max_ nm (∆ε): 233 (−1.94), 276 (−0.28). ESIMS: *m/z* 559 [M+Na]^+^ (Pos.), 535 [M−H]^−^(Neg.). HRESIMS: *m/z*: 559.1788 [M+Na]^+^ (Calcd. for C_26_H_32_O_12_Na, 559.1786). ^1^H-NMR and ^13^C-NMR data see Table 1.

*Matairesinol 4-O-β-d-glucopyranoside* (**3**). White amorphous powder. 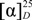 −34.0 (*c* 0.40, MeOH). CD (MeOH) λ_max_ nm (∆ε): 233 (−2.23), 276 (−0.39). ESIMS: *m/z* 543 [M+Na]^+^ (Pos.), 519 [M−H]^−^(Neg.). HRESIMS: *m/z*: 543.1838 [M+Na]^+^ (Calcd. for C_26_H_32_O_11_Na, 543.1837). ^1^H-NMR (DMSO-*d*_6_): *δ*_H_ 6.67 (1H, d, *J* = 1.8 Hz, H-2), 6.96 (1H, d, *J* = 8.3 Hz, H-5), 6.57 (1H, dd, *J* = 8.3, 1.8 Hz, H-6), 2.44–2.48 (3H, m, H-7, 8), 4.05 (1H, m, Ha-9), 3.87 (1H, dd, *J* = 11.2, 4.8 Hz, Hb-9), 6.76 (1H, d, *J* = 1.8 Hz, H-2'), 6.69 (1H, d, *J* = 8.0 Hz, H-5'), 6.60 (1H, dd, *J* = 8.0, 1.8 Hz, H-6'), 2.83 (1H, dd, *J* = 13.5, 5.1 Hz, Ha-7'), 2.73 (1H, m, Hb-7'), 2.69 (1H, m, H-8'), 8.84 (1H, s, 4'-OH), 3.72 (6H, s, 3,3'-OMe), 4.82 (1H, d, *J* = 7.4 Hz, H-1''). ^13^C-NMR (DMSO-*d*_6_): *δ*c 132.6 (C-1), 112.9 (C-2), 148.8 (C-3), 145.1 (C-4), 115.3 (C-5), 120.5 (C-6), 36.9 (C-7), 40.9 (C-8), 70.8 (C-9), 129.0 (C-1'), 113.5 (C-2'), 147.5 (C-3'), 145.1 (C-4'), 115.4 (C-5'), 121.6 (C-6'), 33.8 (C-7'), 45.7 (C-8'), 178.6 (C-9'), 100.2 (C-1''), 73.3 (C-2''), 77.1 (C-3''), 69.7 (C-4''), 76.9 (C-5''), 60.7 (C-6''), 55.6 (C-3, 3'-OMe).

*Nortrachelogenin 4,4'-di-O-β-d-glucopyranoside* (**4**). White amorphous powder. ESIMS: *m/z* 716 [M+NH_4_]^+^, 721 [M+Na]^+^ (Pos.), 697 [M−H]^−^ (Neg.). ^1^H-NMR (DMSO-*d*_6_): *δ*_H_ 6.72 (1H, d, *J* = 1.7 Hz, H-2), 6.99 (1H, d, *J* = 8.5 Hz, H-5), 6.74 (1H, dd, *J* = 8.5, 1.7 Hz, H-6), 2.65 (1H, m, Ha-7), 2.46 (br d, *J* = 10.1 Hz, Hb-7), 2.40 (1H, m, H-8), 3.97 (2H, br d, *J* = 8.3 Hz, H-9), 6.83 (1H, d, *J* = 1.6 Hz, H-2'), 6.97 (1H, d,*J* = 8.3 Hz, H-5'), 6.62 (1H, dd, *J* = 8.3, 1.6 Hz, H-6'), 3.02 (1H, d, *J* = 13.5 Hz, Ha-7'), 2.88 (1H, d, *J* = 13.5 Hz, Hb-7'), 6.30 (1H, s, 8'-OH), 3.70, 3.73 (each 3H, s, 3, 3'-OMe), 4.85, 4.82 (each 1H, d, *J* = 7.5 Hz, H-1'', H-1'''). ^13^C-NMR (DMSO-*d*_6_): *δ*c 132.9 (C-1), 129.2 (C-1'), 113.0 (C-2), 114.8 (C-2'), 148.4, 148.8 (C-3, 3'), 145.1, 145.5 (C-4, 4'), 115.3 (C-5, 5'), 120.5 (C-6), 122.5 (C-6'), 30.9 (C-7), 42.8 (C-8), 70.0 (C-9), 75.4 (C-8'), 178.0 (C-9'), 100.2, 100.0 (C-1'', 1'''), 73.2 (C-2'', 2'''), 77.0 (C-3'', 3'''), 69.7 (C-4'', 4'''), 76.9 (C-5'', 5'''), 60.6, 60.7 (C-6'', 6'''), 55.6, 55.7 (C-3, 3'-OMe).

### 3.5. Cell Lines and Reagents

HepG2/STAT1 (HepG2/STAT3) cells, a gift from Prof. Xinyuan Fu (National University of Singapore, Singapore, Singapore), were a human hepatoma derived cell line cells stably transfected with a STAT1 (STAT3)-responsive firefly luciferase reporter plasmid. Luciferase assay kit was purchased from Promega Corp., Madison, WI, USA. Recombinant human IFN-γ was purchased from Shanghai Clone High Biological Technology Co Ltd., Shanghai, China. Recombinant human IL-6 was purchased from Pepprotech Inc., Rocky Hill, NJ, USA. Pyridone 6 and AG490 were purchased from Merck Biosciences, Darmstadt, Germany and Calbiochem, San Diego, CA, USA, respectively.

### 3.6. Luciferase Assay

Luciferase assay was performed as previous described [32]. HepG2/STAT1 (HepG2/STAT3) cells (2 × 10^4^ per well) maintained in a DMEM medium were seeded into 96-well cell culture microplates and allowed to grow for 24 h at 37 °C in a 5% CO_2_ incubator, and then treated with test samples for 1h followed by stimulation with 50 IU/mL IFN-γ (10 ng/mL IL-6) for 6 h. Luciferase activity was determined using the Promega luciferase kits according to the manufacturer’s instruction. The cell number was counted at seeding and controlled by equal seeding. All luciferase assays were repeated at least twice to minimize the variance caused by cell number. IFN-γ (IL-6)-induced STAT1 (STAT3)-responsive luciferase activity (% of control) was calculated as {[Fluorescence intensity with sample and IFN-γ (IL-6) treatment − Fluorescence intensity without IFN-γ (IL-6) treatment]/[Fluorescence intensity with IFN-γ (IL-6) treatment − Fluorescence intensity without IFN-γ (IL-6) treatment]} × 100.

## 4. Conclusions

In summary, two new dibenzylbutyrolactone lignans, 4-demethyltraxillaside (**1**) and nortrachelogenin 4-*O*-*β*-d-glucopyranoside (**2**), together with six known compounds were isolated from Caulis Trachelospermi. Their structures and stereochemistries were established by the analysis of 1D, 2D-NMR, MS, OR and CD spectra. The extract of Caulis Trachelospermi and its main dibenzylbutyrolactone lignan components had strong inhibiting activity on JAK/STAT pathway, and it may be an important mechanism for the reported anticancer and anti-inflammation function of Caulis Trachelospermi. However, it is still unclear whether the inhibition of IFN-γ/STAT1 and IL-6/STAT3 signaling pathways by the active components is direct on these pathways or whether it occurs via the upregulation of the expression of other factors that then inhibit these signaling pathways. Further research is needed for the determination of their precise target.

## Supplementary Materials

Supplementary materials can be accessed at: http://www.mdpi.com/1420-3049/19/8/11560/s1.

## Supplementary Files

Supplementary File 1
