# Supplementary material for: Active Components with Inhibitory Activities on IFN-γ/STAT1 and IL-6/STAT3 Signaling Pathways from Caulis Trachelospermi
Source: Molecules. 2014 Aug 5;19(8):11560–71. doi: 10.3390/molecules190811560 (PMC6270818; doi:10.3390/molecules190811560)

Supplementary Materials

In this "Supplementary Materials" file for the manuscript, "Active Components with Inhibitory Activities on IFN-γ/STAT1 and IL-6/STAT3 Signaling Pathways from Caulis Trachelospermi", ^{1}H-NMR, ^{13}C-NMR, HMQC, HMBC, ESIMS and HRESIMS spectra of compounds **1**–**3** as well as ^{1}H-NMR, ^{13}C-NMR and ESIMS of compound **4** are available here as listed below.

Table S1. 400 MHz ^{1}H- and 100 MHz ^{13}C-NMR data of **1** in DMSO-d_6 (δ in ppm, J in Hz) [a].

Position	δ_C	δ_H	HMBC
1	128.8		
2	106.0	6.34 (1H, s)	C-3, C-4, C-6, C-7
3	148.0		
4	133.9		
5	148.0		
6	106.0	6.34 (1H, s)	C-2, C-4, C-5, C-7
7	37.3	2.45–2.52 (2H, m)	C-1, C-2, C-6, C-8, C-9
8	40.9	2.40–2.45 (1H, m)	
9	70.8	Ha 4.11 (1H, dd, 8.5, 7.2)<br>Hb 3.89 (1H, t, 8.5)	C-9'<br>C-7
1'	131.9		
2'	113.9	6.80 (1H, d, 1.8)	C-3', C-4', C-6', C-7'
3'	148.7		
4'	145.4		
5'	115.1	6.99 (1H, d, 8.3)	C-1', C-3', C-4'
6'	121.4	6.68 (1H, dd, 8.3, 1.8)	C-2', C-4', C-7'
7'	33.6	2.80–2.83 (2H, m)	C-1', C-2', C-6', C-8', C- 9'
8'	45.6	2.75 (1H, dd, 8.2, 6.1)	C- 9'
9'	178.6		
1''	100.3	4.84 (1H, d, 7.3)	C-4'
2''	73.3		
3''	77.0		
4''	69.7		
5''	76.9		
6''	60.7		
4-OH		8.17 (1H, s)	
3-OCH_3	56.0	3.71 (3H, s)	C-3
3'-OCH_3	55.7	3.72 (3H, s)	C-3'
5-OCH_3	56.0	3.71 (3H, s)	C-5

[a] Signals assignments were based on the results of HMQC, and HMBC experiments.

Table S2. 400 MHz ^{1}H- and 100 MHz ^{13}C-NMR data of **2** in DMSO-d_6 (δ in ppm, J in Hz) [a].

Position	δ_C	δ_H	HMBC
1	133.0		
2	113.0	6.70 (1H, d, 1.6)	C-1, C-4, C-6, C-7
3	148.8		
4	145.1		
5	115.3	6.97 (1H, d, 8.3)	C-1, C-3, C-4
6	120.5	6.61 (1H, br d, 8.3)	C-2, C-4
7	30.9	Ha 2.61 (1H, dd, 12.3, 2.4)	
		Hb 2.44 (1H, br d, 12.3)	
8	42.8	2.39 (1H, m)	
9	70.0	3.95 (2H, d, 7.7)	C-8, C-8', C-9'
1'	126.4		
2'	114.5	6.77 (1H, br s)	C-4', C-6', C-7'
3'	147.2		
4'	145.4		
5'	115.3	6.68 (1H, d, 8.0)	C-1', C-3'
6'	122.7	6.61 (1H, br d, 8.0)	C-2', C-4'
7'	40.0 (overlapped)	Ha 2.98 (1H, d, 13.8)	C-1', C-2', C-6', C-8'
		Hb 2.83 (1H, d, 13.8)	C-1', C-2', C-6', C-8', C-9'
8'	75.4		
9'	178.1		
1''	100.2	4.82 (1H, d, 7.2)	C-4'
2''	73.2		
3''	77.0		
4''	69.7		
5''	76.9		
6''	60.7		
4'-OH		8.85 (1H, s)	
8'-OH		6.21 (1H, s)	
3-OCH_3	55.6	3.70 (3H, s)	C-3
3'-OCH_3	55.6	3.73 (3H, s)	C-3'

[a] Signals assignments were based on the results of HMQC, and HMBC experiments.

Table S3. 400 MHz ^{1}H- and 100 MHz ^{13}C-NMR data of **3** in DMSO-d_6 (δ in ppm, J in Hz) [a].

Position	δ_C	δ_H	HMBC
1	132.6		
2	112.9	6.67 (1H, d, 1.8)	C-4, C-6, C-7
3	148.8		
4	145.1		
5	115.3	6.96 (1H, d, 8.3)	C-1, C-3, C-4
6	120.5	6.57 (1H, dd, 8.3, 1.8)	C-2, C-4, C-7
7	36.9	2.44–2.48 (2H, m)	C-1, C-2, C-6, C-8
8	40.9	2.44–2.48 (1H, m)	
9	70.8	Ha 4.05 (1H, m)	C-9'
		Hb 3.87 (1H, dd, 11.2, 4.8)	C-7, C-8

Table S3. *Cont.*

Position	δ_C	δ_H	HMBC
1'	129.0		
2'	113.5	6.76 (1H, d, 1.8)	C-4', C-6', C-7'
3'	147.5		
4'	145.1		
5'	115.4	6.69 (1H, d, 8.0)	C-1', C-3'
6'	121.6	6.60 (1H, dd, 8.0, 1.8)	C-2', C-4', C-7'
7'	33.8	Ha 2.83 (1H, dd, 13.3, 5.1)	C-1', C-2', C-6', C-8', C-9', C-8
		Hb 2.73 (1H, m)	C-1', C-2', C-6', C-8', C-9', C-8
8'	45.7	2.69 (1H, m)	C-8
9'	178.6		
1''	100.2	4.82 (1H, d, 7.4)	C-4'
2''	73.3		
3''	77.1		
4''	69.7		
5''	76.9		
6''	60.7		
4'-OH		8.84 (1H, s)	
3-OCH_3	55.6	3.72 (3H, s)	C-3
3'-OCH_3	55.6	3.72 (3H, s)	C-3'

[a] Signals assignments were based on the results of HMQC, and HMBC experiments.

Apendix of Spectra

SP1:

Figure S1. Positive (**A**) and negative (**B**) ESIMS spectra of **1**.

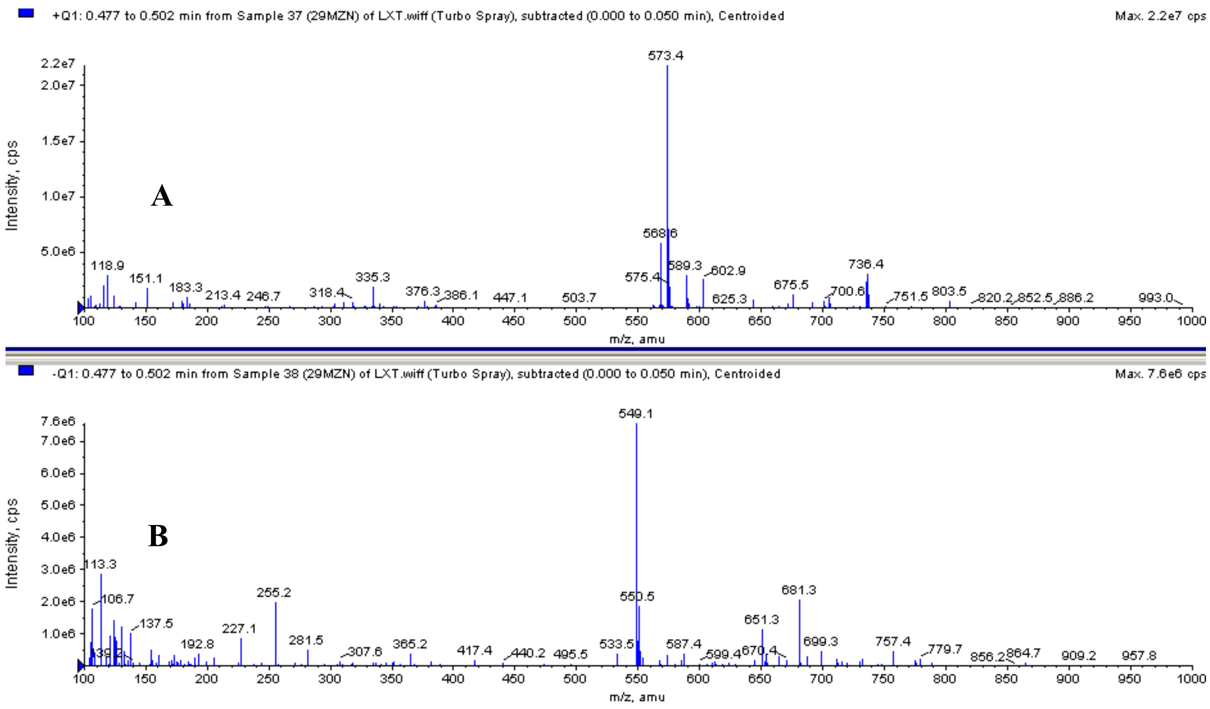

Figure S2. Positive HRESIMS spectrum of **1**.

Qualitative Analysis Report

Data Filename	328.d	Sample Name	LXL-14
Instrument Name	TOF G6230A	Acquired Time	2014.05.26
Acq Method	YCL.M	Acquired SW	6200 series TOF/6500 series
IRM Calibration Status	Success		

User Chormatograms

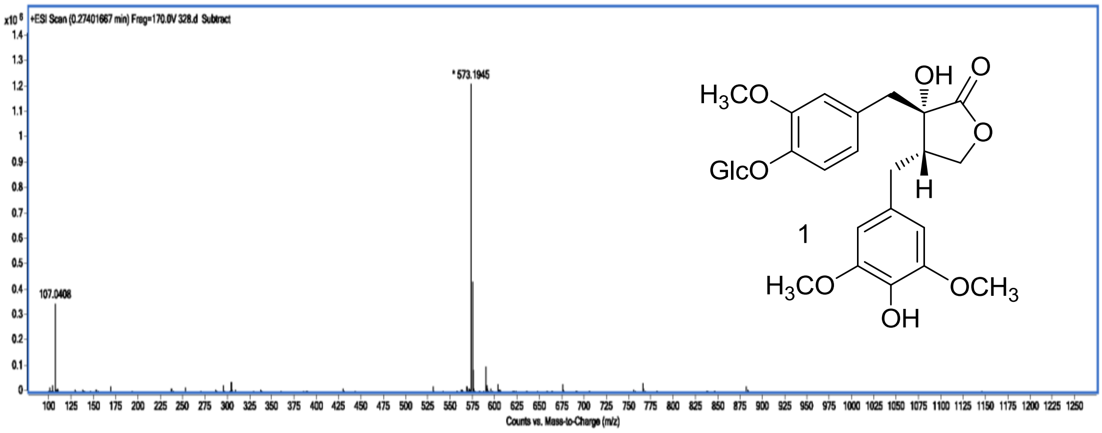

Figure S3. 400 MHz ^{1}H-NMR spectrum of **1** in DMSO-d_6.

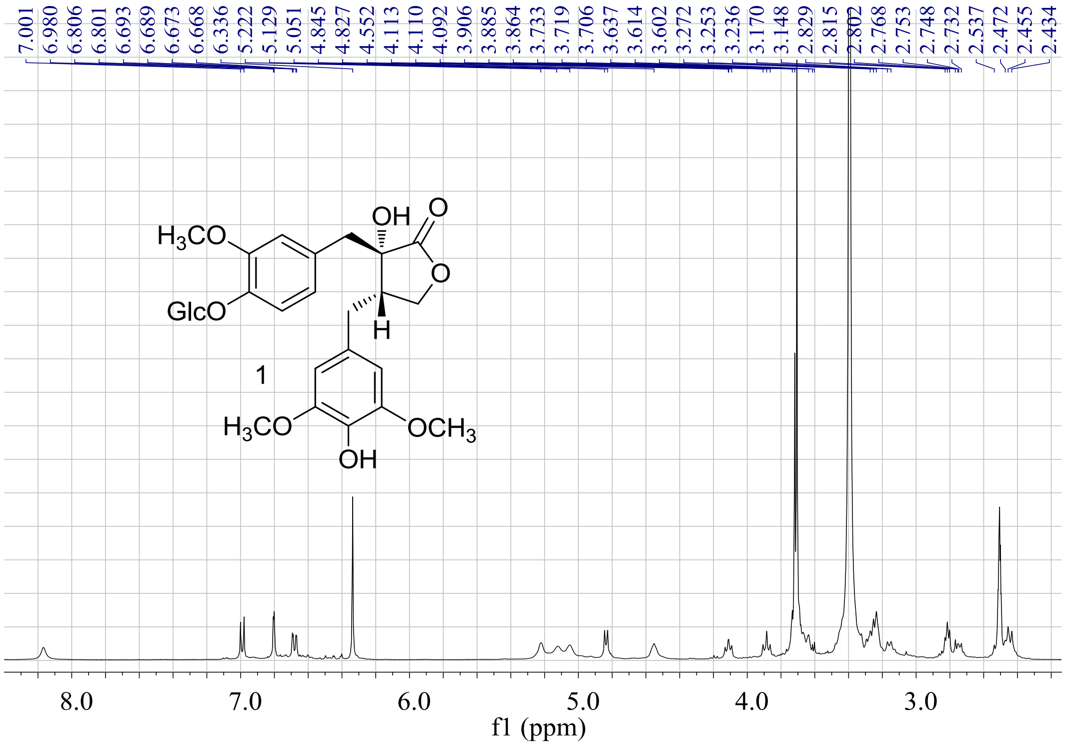

Figure S4. 100 MHz ^{13}C-NMR spectrum of **1** in DMSO-d_6.

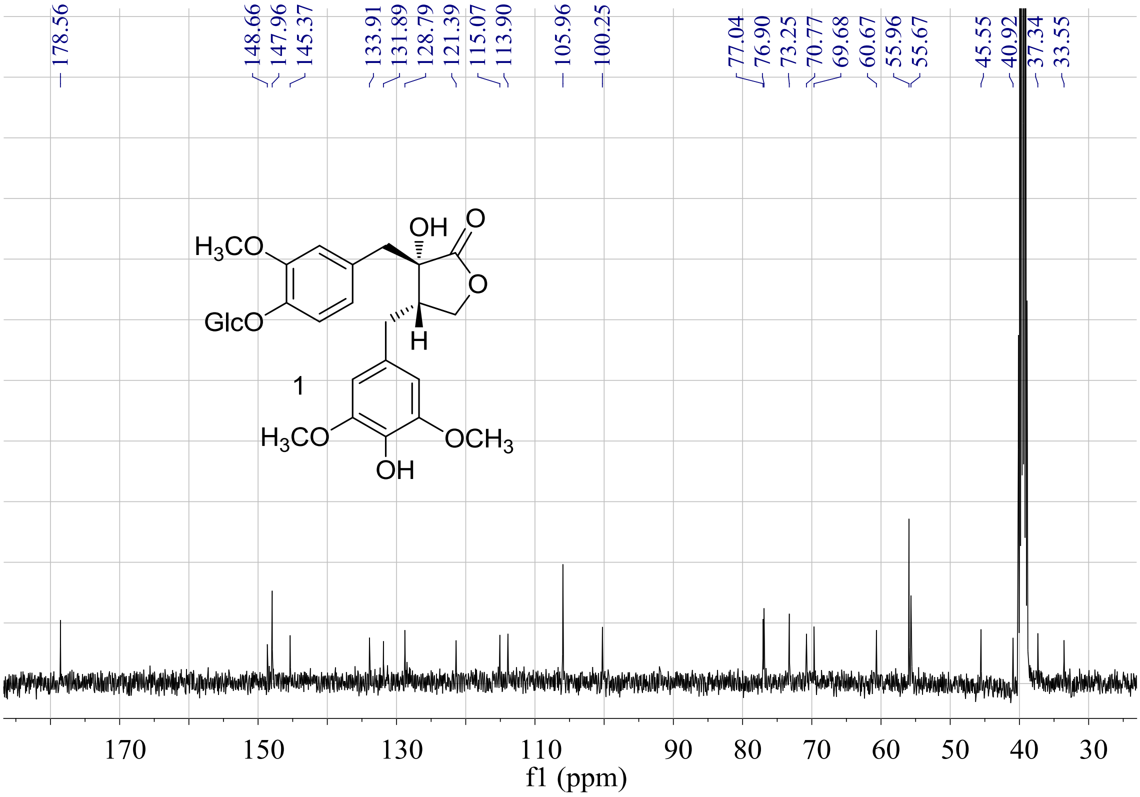

Figure S5. HMQC spectrum of **1** in DMSO-d_6.

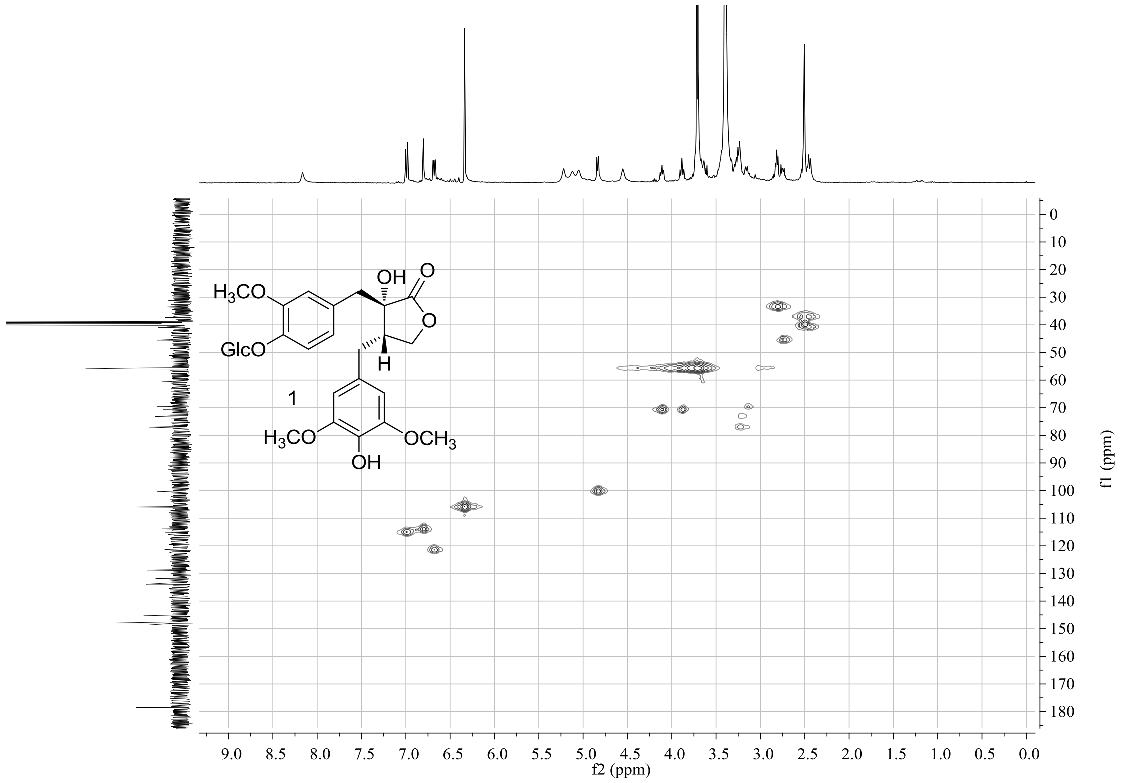

Figure S6. HMBC spectrum of **1** in DMSO-d_6.

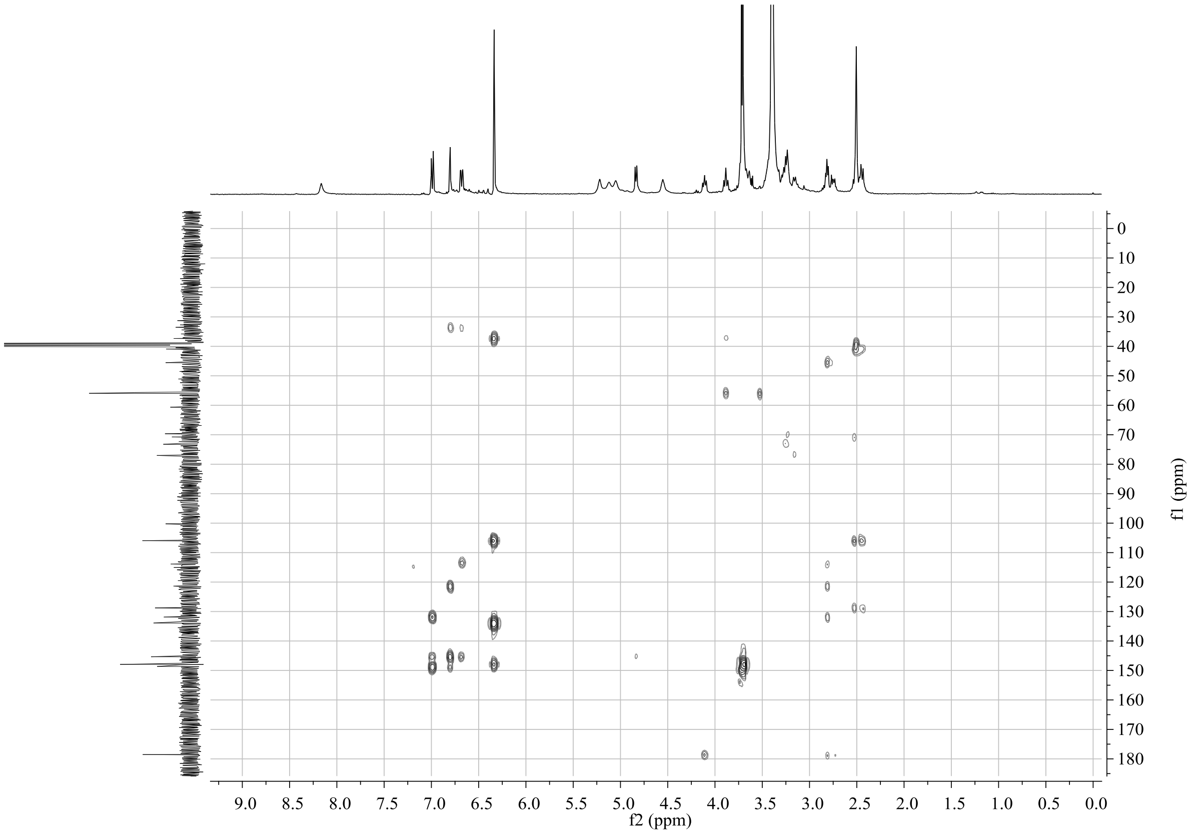

SP2:

Figure S7. Positive (**A**) and negative (**B**) ESIMS spectra of **2**.

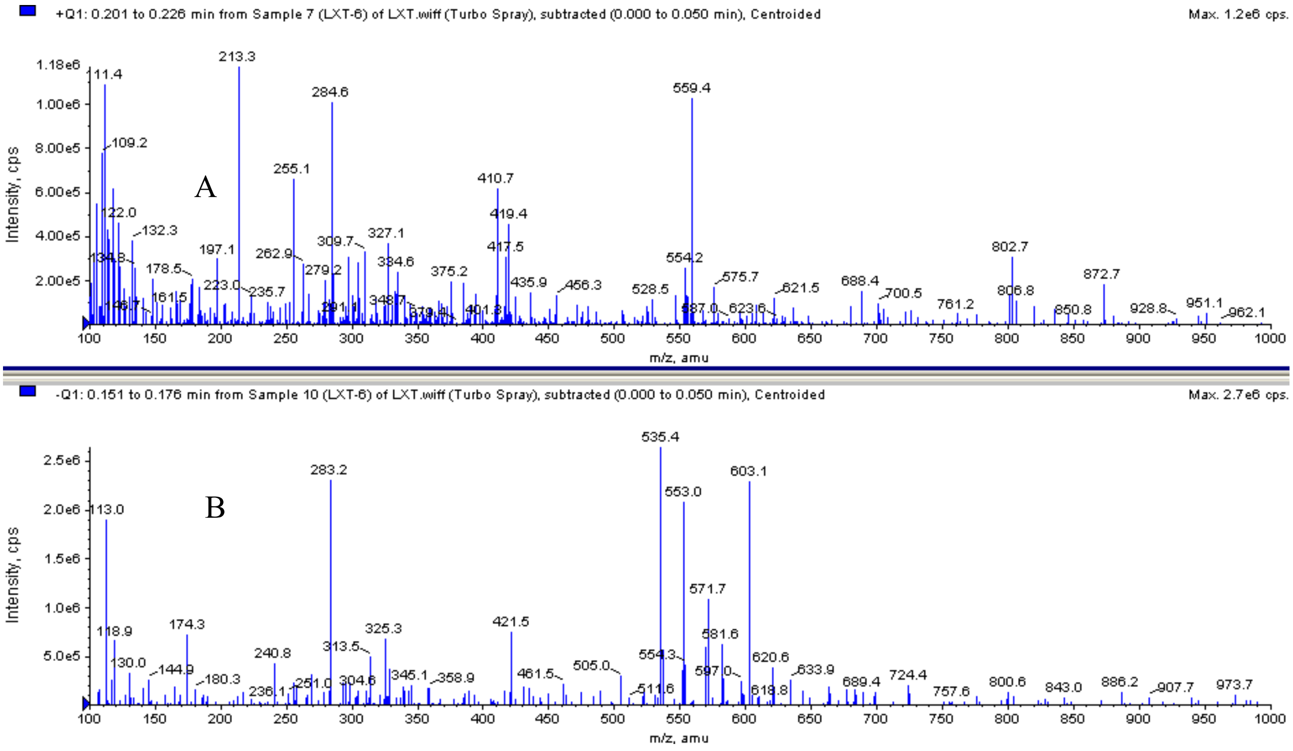

Figure S8. Positive HRESIMS spectrum of **2**.

Qualitative Analysis Report

**Data Filename**	261-3.D	**Sample Name**	LXT-6
**Instrument Name**	TOF G6230A	**Acquired Time**	2014.04.28
**Acq Method**	YCL.M	**Acquired SW**	6200 series TOF/6500 series
**IRM Calibration Status**	Success		

User Chormatograms

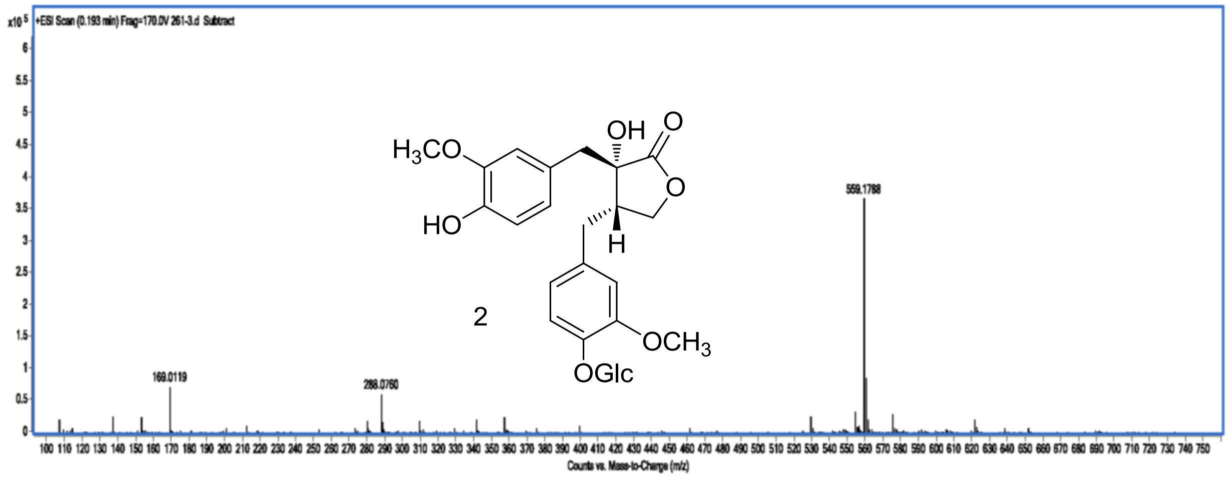

Figure S9. 400 MHz ^{1}H-NMR spectrum of **2** in DMSO-d_6.

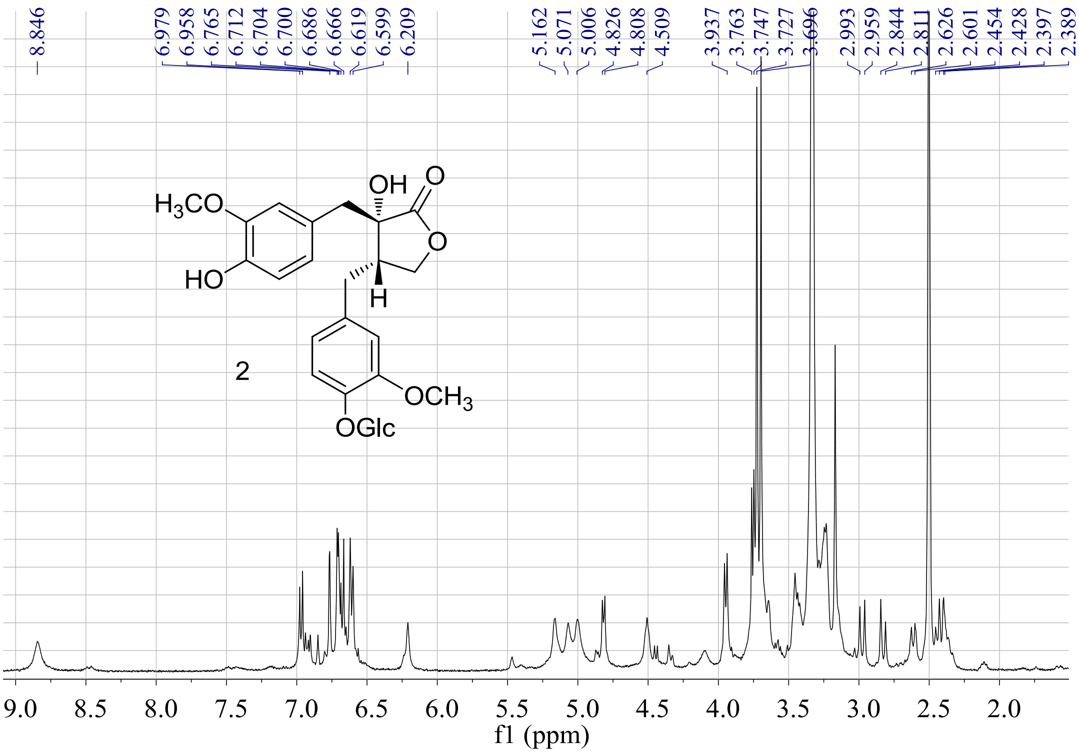

Figure S10. 100 MHz ^{13}C-NMR spectrum of **2** in DMSO-d_6.

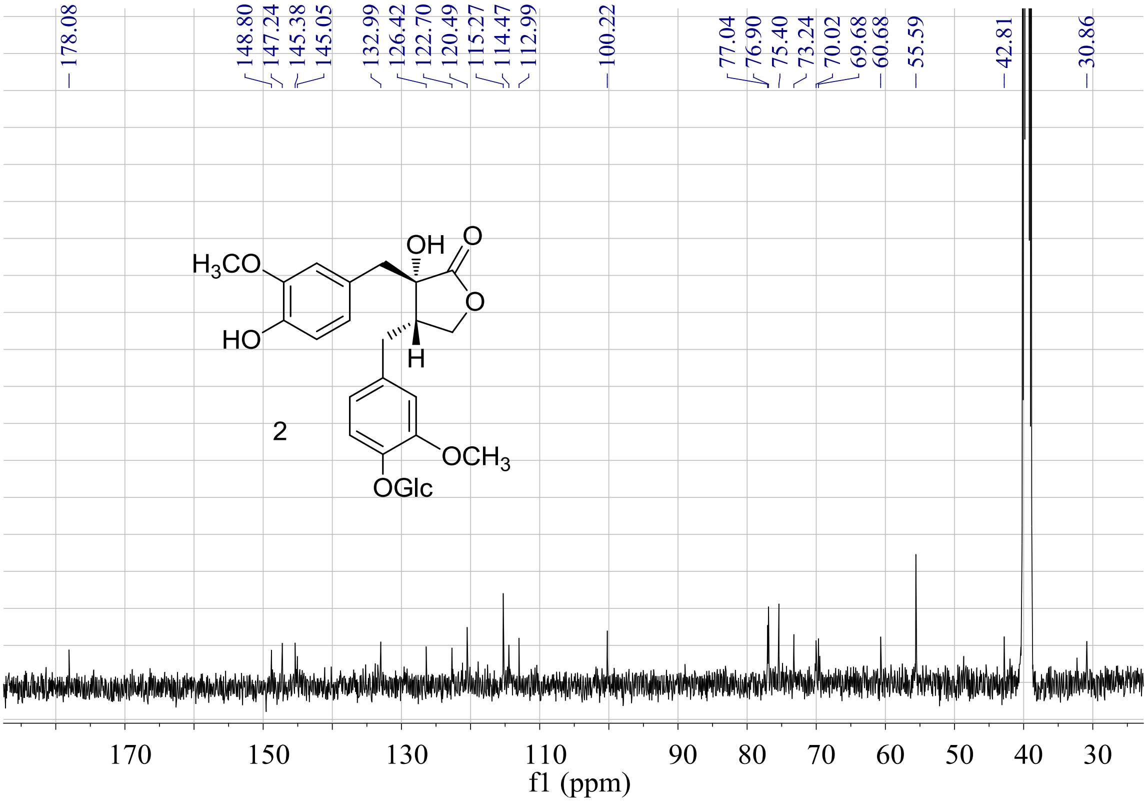

Figure S11. HMQC spectrum of **2** in DMSO-d_6.

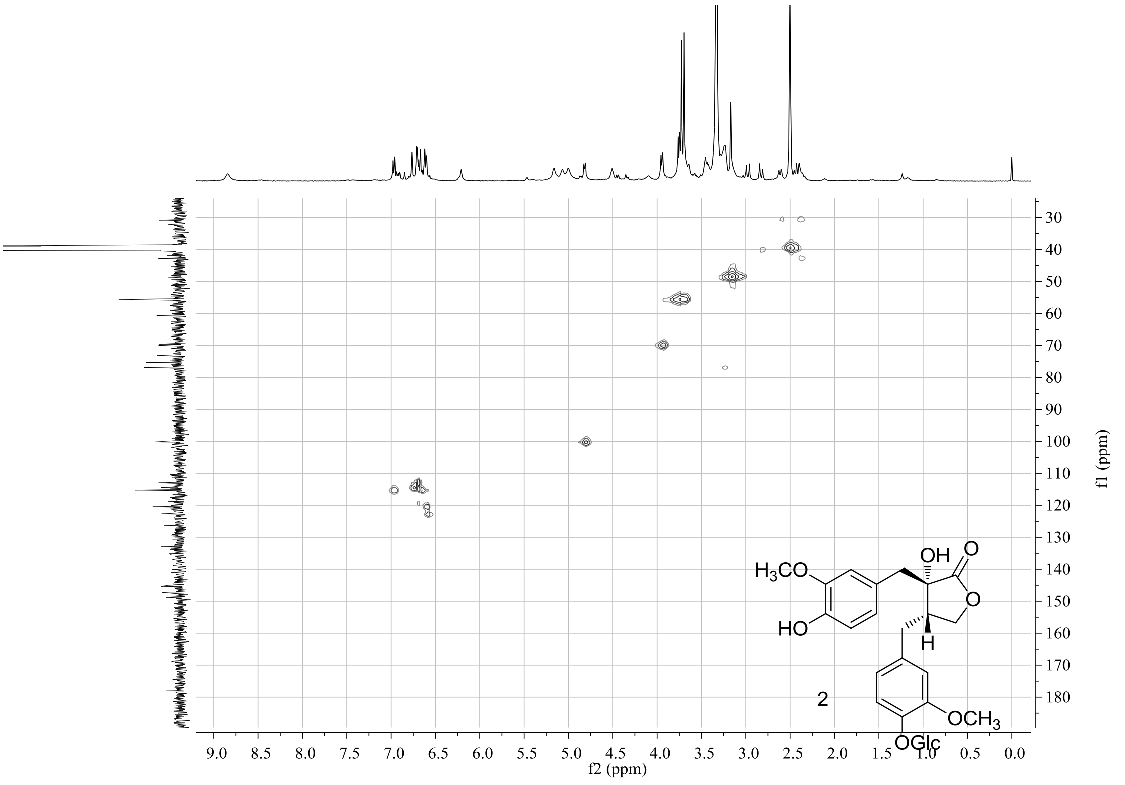

Figure S12. HMBC spectrum of **2** in DMSO-d_6.

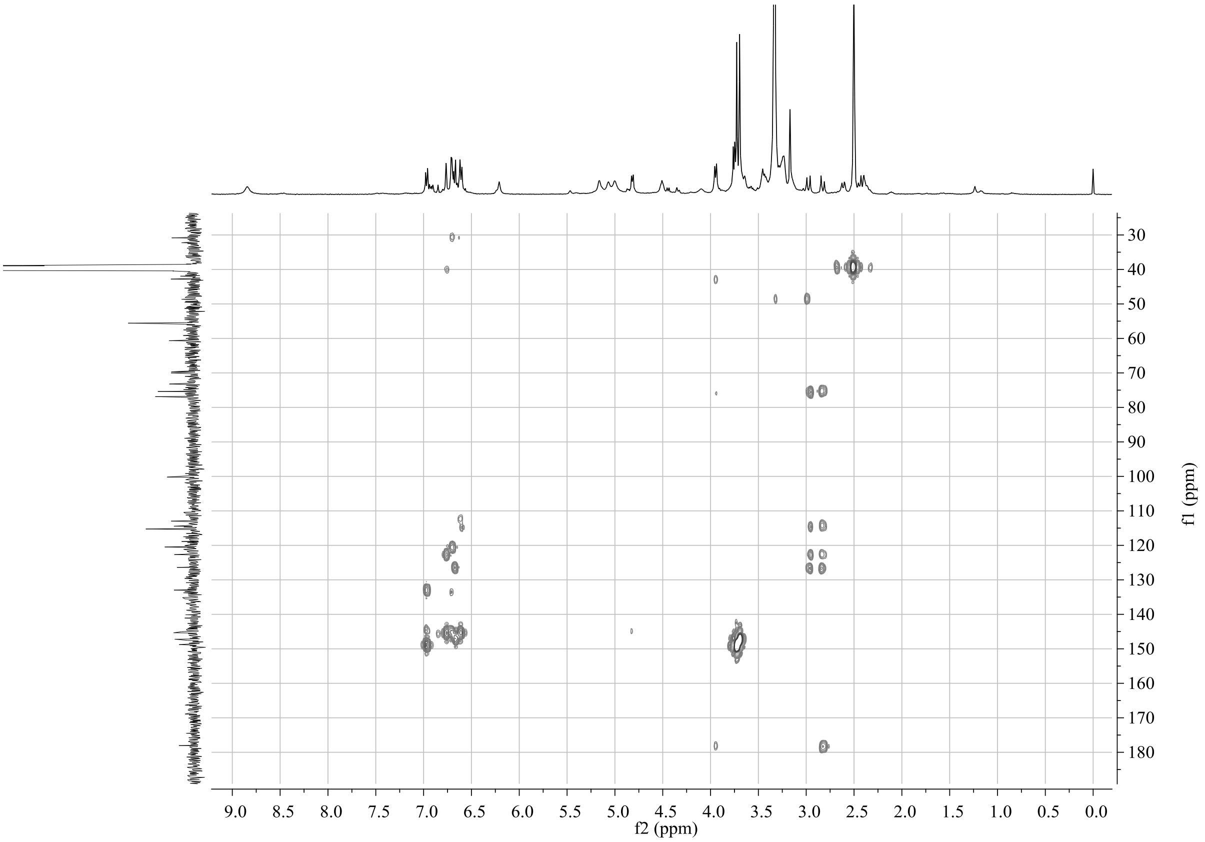

SP3:

Figure S13. Positive (**A**) and negative (**B**) ESIMS spectra of **3**.

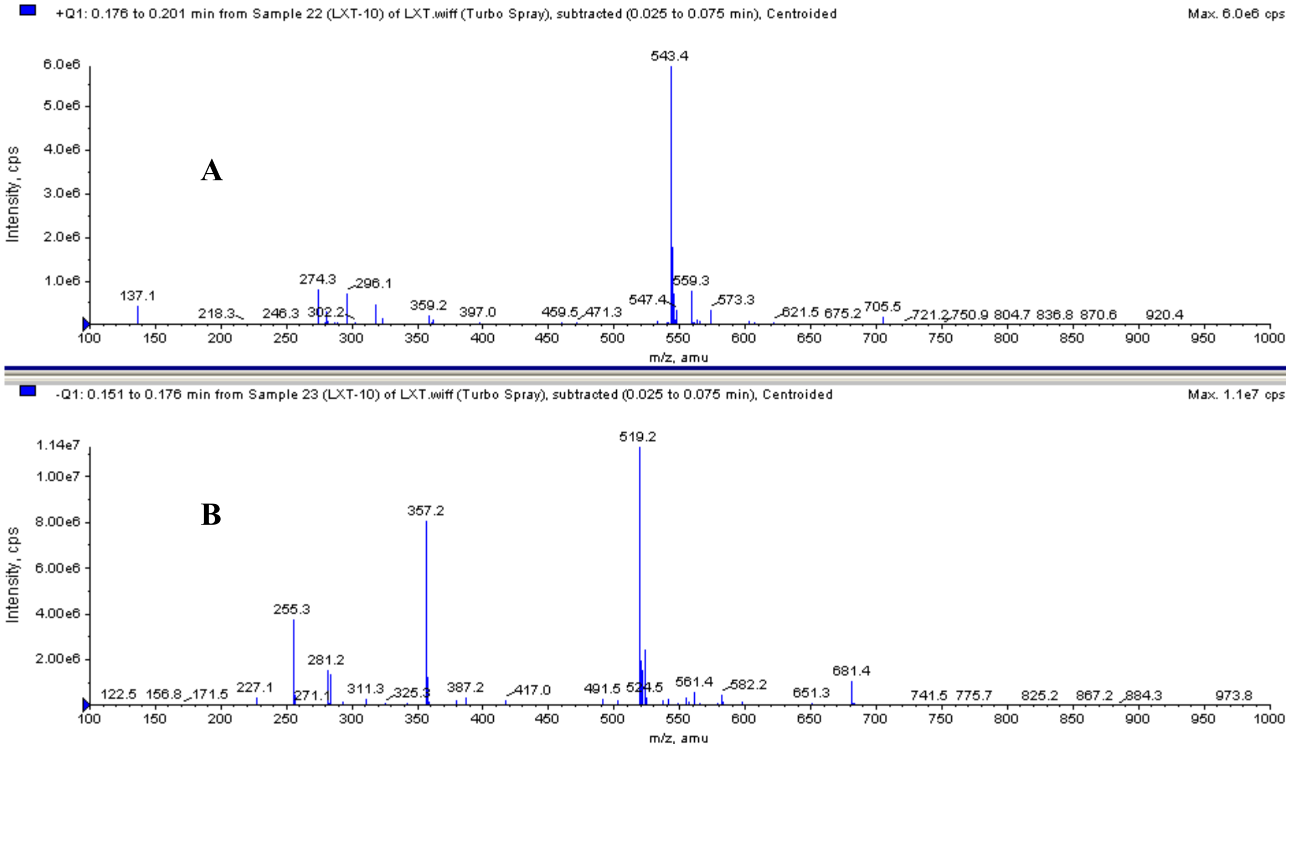

Figure S14. Positive HRESIMS spectrum of **3**.

Qualitative Analysis Report

**Data Filename**	262-3.D	**Sample Name**	LXT-10
**Instrument Name**	TOF G6230A	**Acquired Time**	2014.04.28
**Acq Method**	YCL.M	**Acquired SW**	6200 series TOF/6500 series
**IRM Calibration Status**	Success		

User Chormatograms

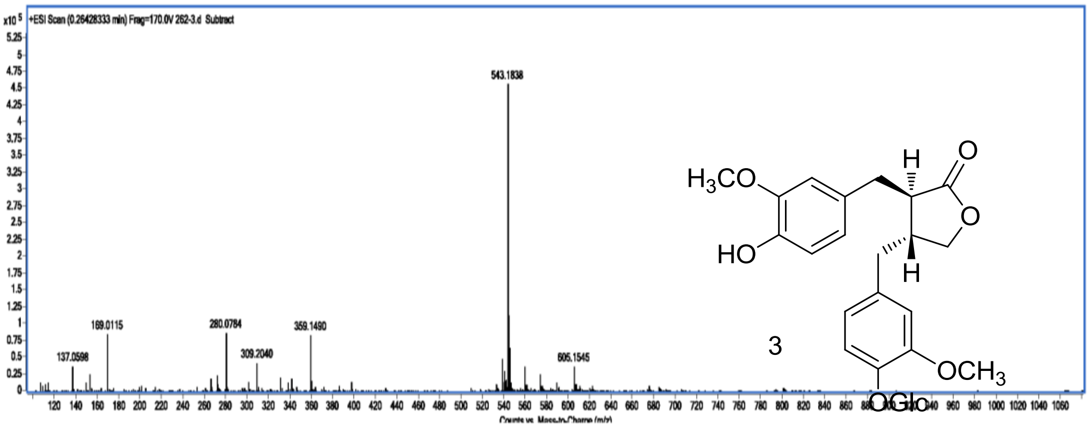

Figure S15. 400 MHz ^{1}H-NMR spectrum of **3** in DMSO-d_6.

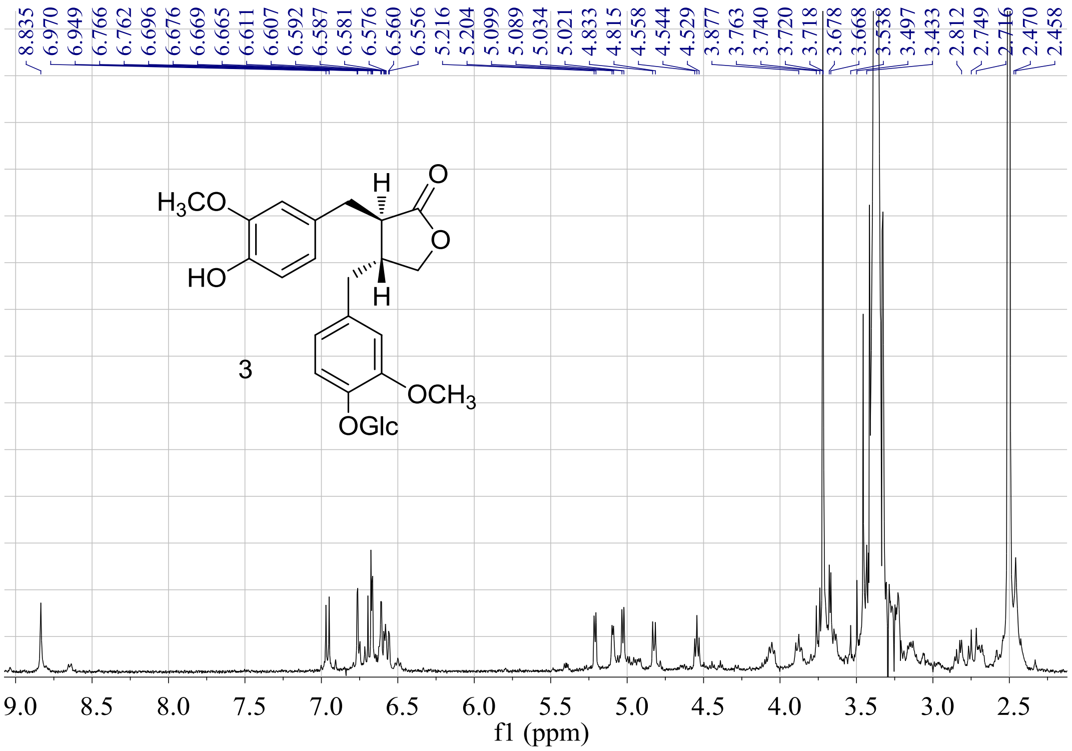

Figure S16. 100 MHz ^{13}C-NMR spectrum of **3** in DMSO-d_6.

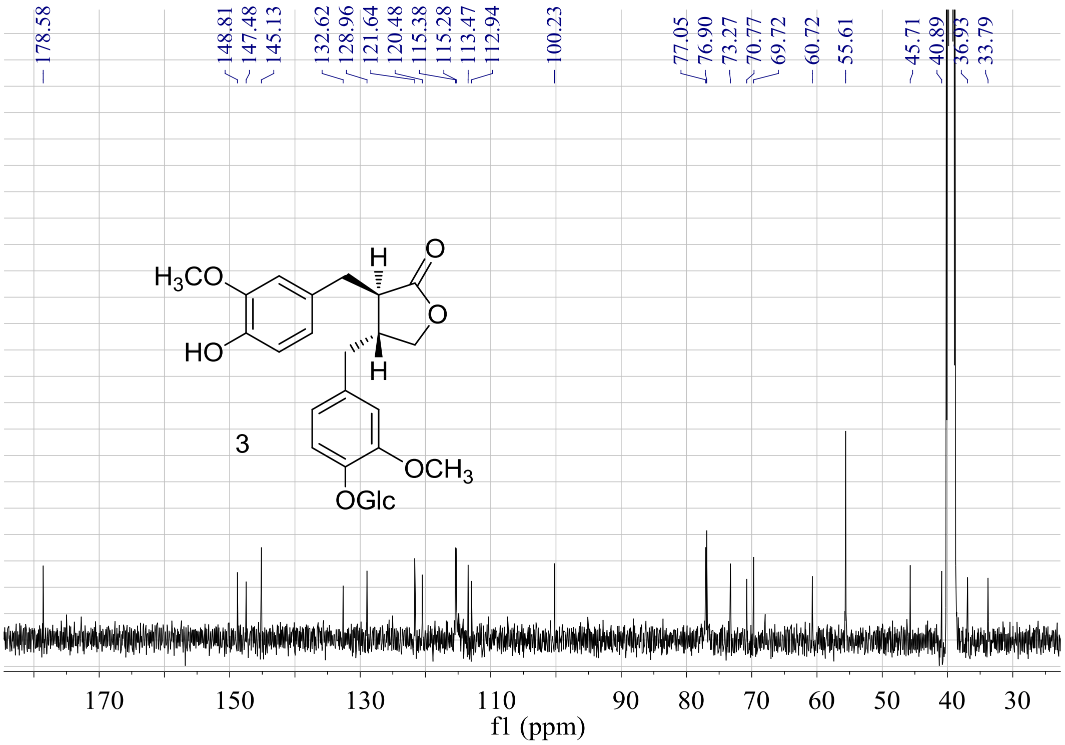

Figure S17. HMQC spectrum of **3** in DMSO-d_6.

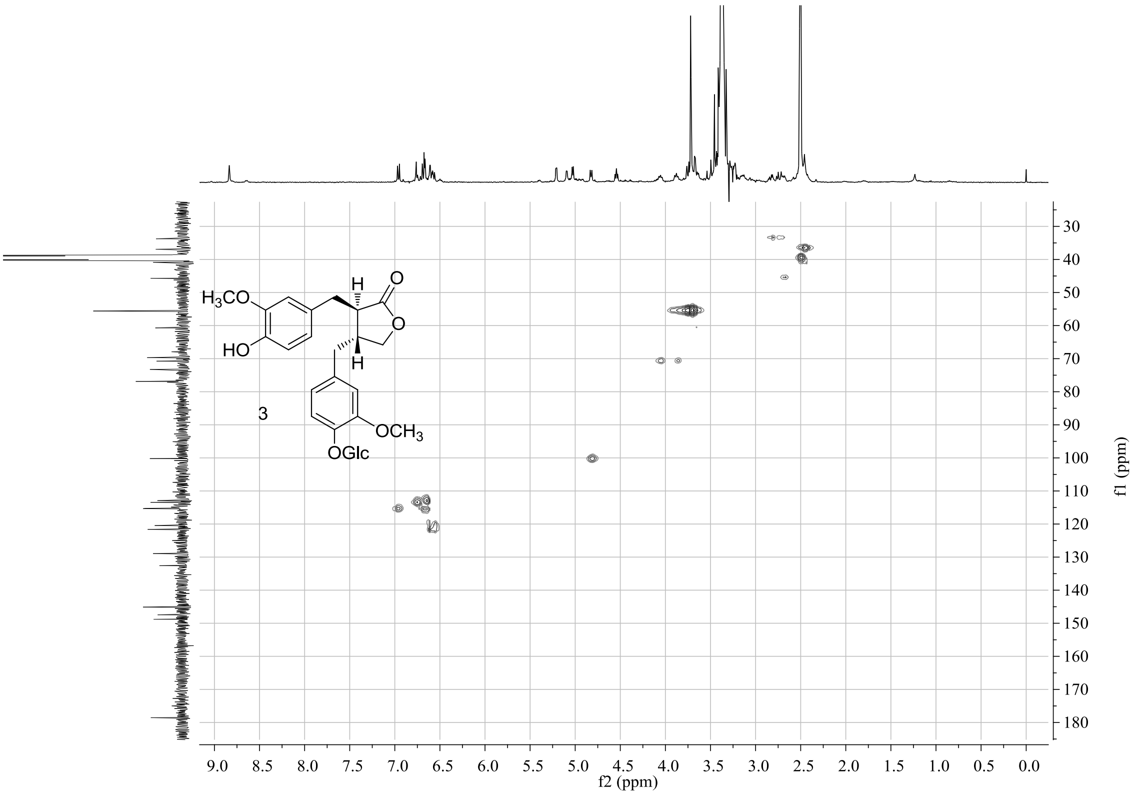

Figure S18. HMBC spectrum of **3** in DMSO-d_6.

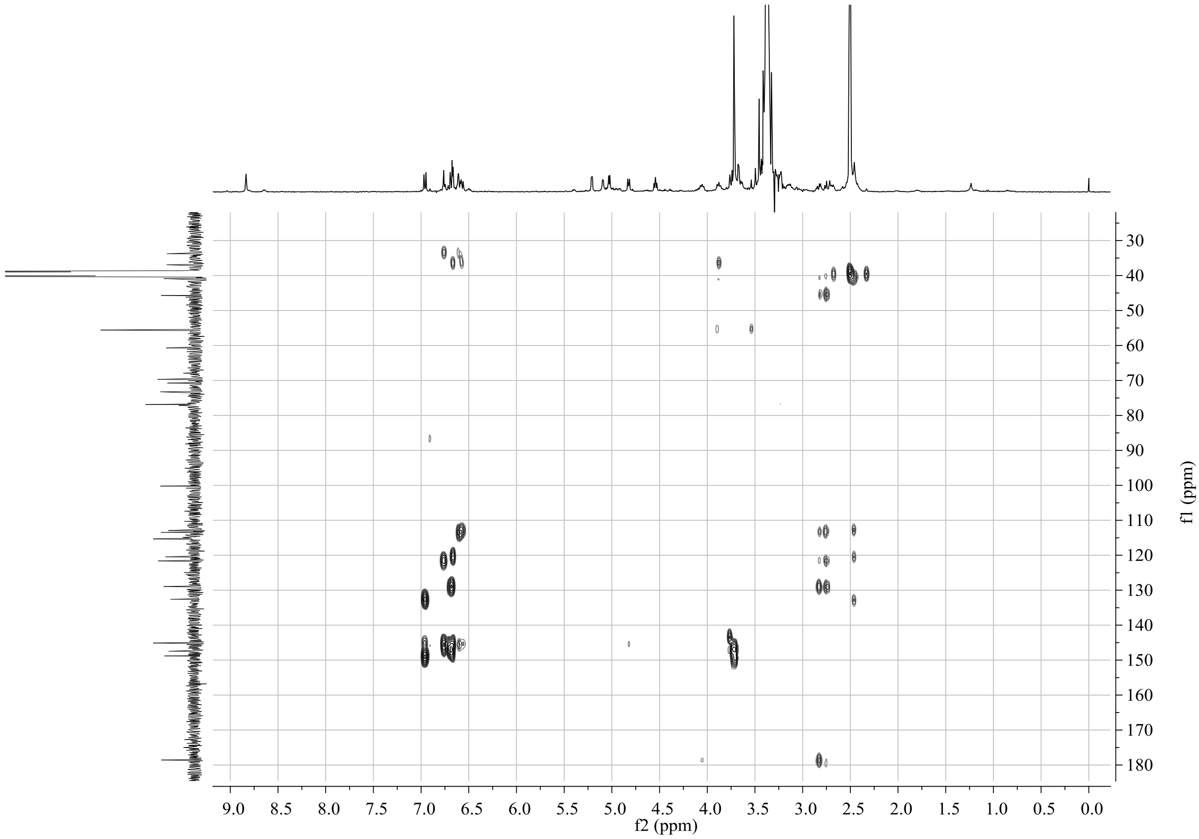

SP4:

Figure S19. Positive (**A**) and negative (**B**) ESIMS spectra of **4**.

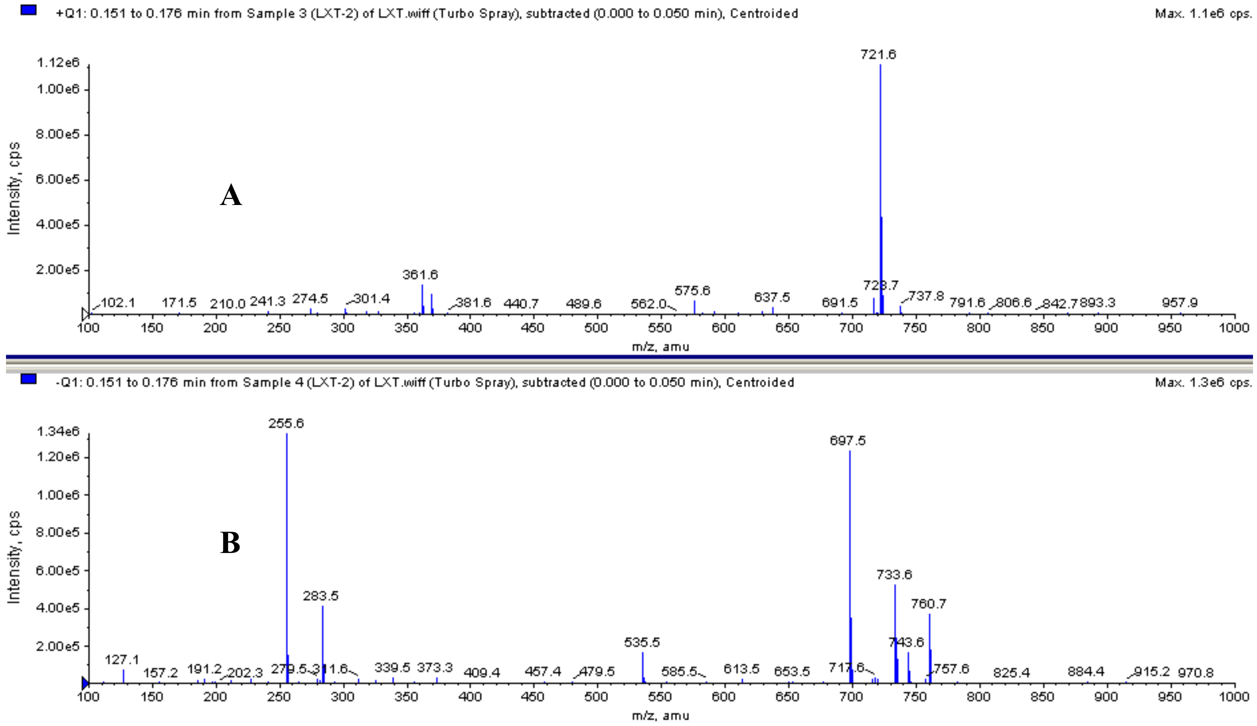

Figure S20. 400 MHz ^{1}H-NMR spectrum of **4** in DMSO-d_6.

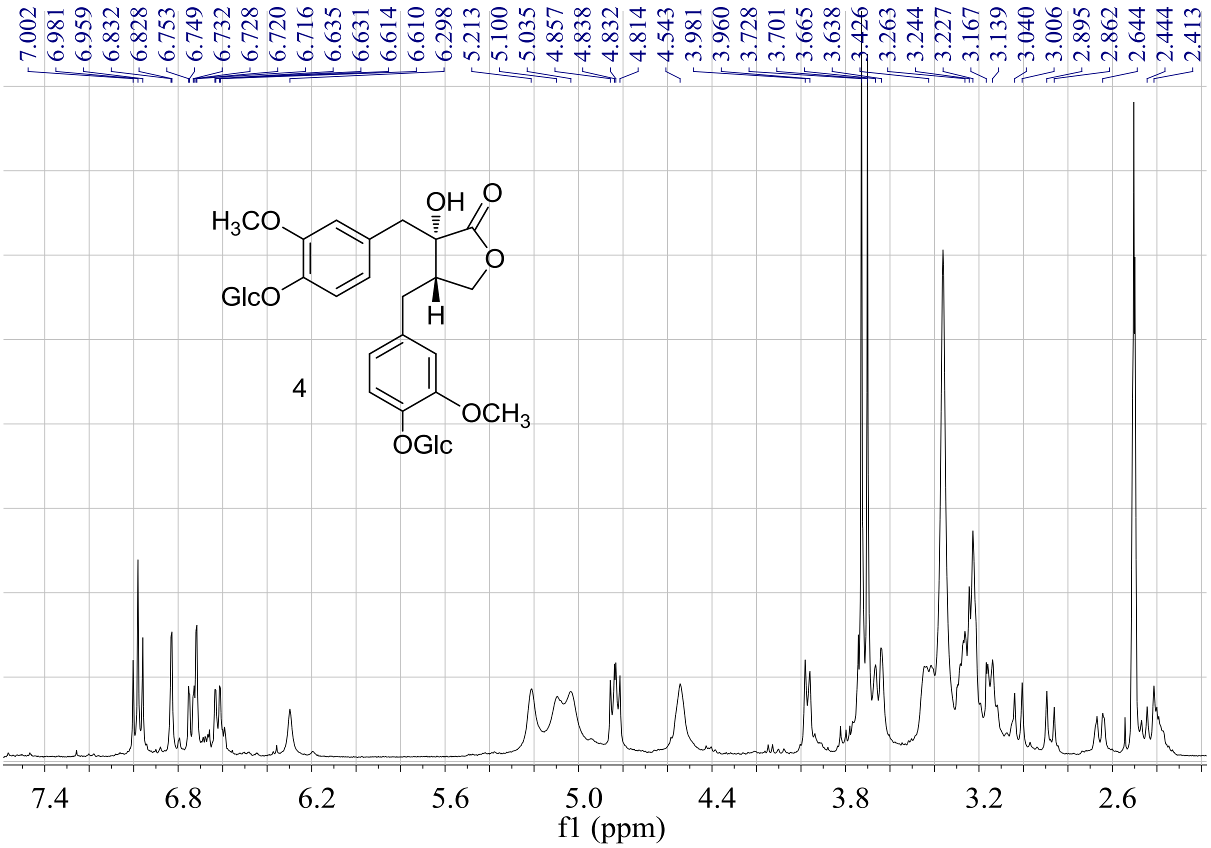

Figure S21. 100 MHz ^{13}C-NMR spectrum of **4** in DMSO-d_6.

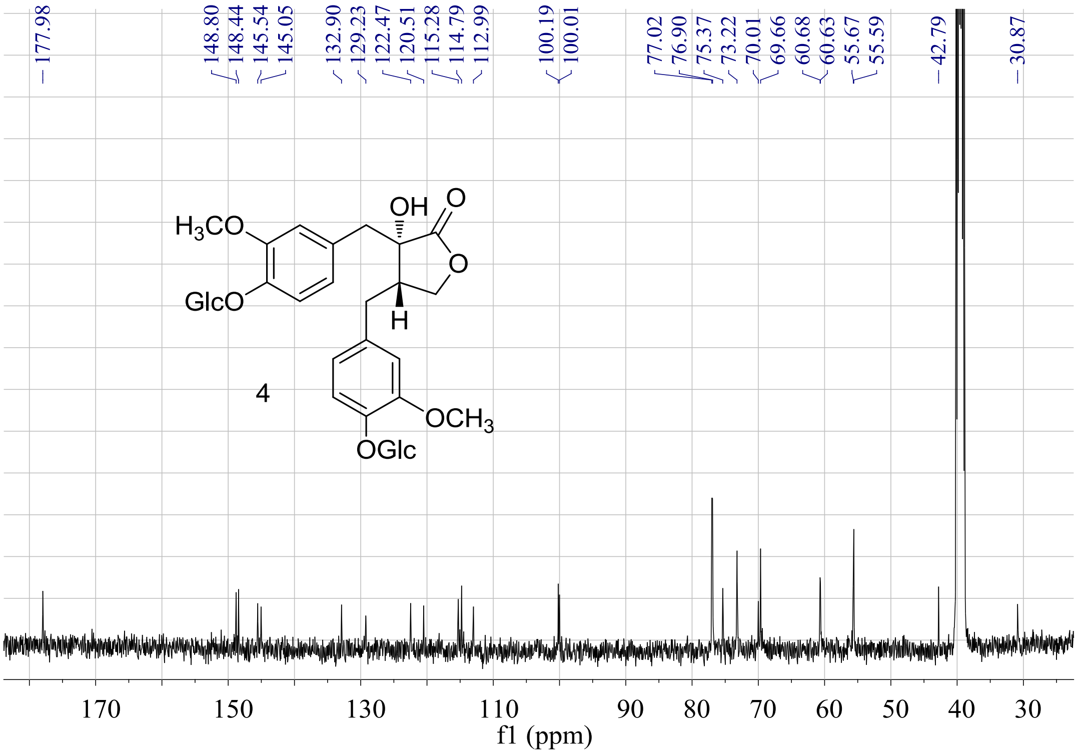

Supplement: Supplementary File 1 [file molecules-19-11560-s001.pdf]
